# Biomimetic Synthesis of Silver Nanoparticles Using Ethyl Acetate Extract of *Urtica diocia* Leaves; Characterizations and Emerging Antimicrobial Activity

**DOI:** 10.3390/microorganisms10040789

**Published:** 2022-04-08

**Authors:** Mohammed Binsalah, Sandhanasamy Devanesan, Mohamad S. AlSalhi, Abdullrahman Nooh, Osama Alghamdi, Nasser Nooh

**Affiliations:** 1 Department of Oral and Maxillofacial Surgery, College of Dentistry, King Saud University, Riyadh 11545, Saudi Arabia; masalah@ksu.edu.sa (M.B.); oghamdi@ksu.edu.sa (O.A.); nnooh@ksu.edu.sa (N.N.); 2 Research Chair in Laser Diagnosis of Cancers, Department of Physics and Astronomy, College of Science, King Saud University, Riyadh 11451, Saudi Arabia; 3 Department of Internal Medicine, Prince Mohammed Bin Abdulaziz Hospital, Riyadh 7333, Saudi Arabia; nooh29@gmail.com

**Keywords:** UD-AgNPs, characterization, green synthesis, antimicrobial effects

## Abstract

The current work reports the biosynthesis of silver nanoparticles (AgNPs) using the antimicrobial activities of ethyl acetate extract of *Urtica diocia* (UD) leaves as a reducing and capping agent. The synthesized UD-AgNPs were characterized using UV–visible spectroscopy, scanning electron microscopy (SEM), transmission electron microscopy (TEM), energy-dispersive X-ray analysis (EDAX), Fourier transform infrared (FTIR) spectroscopy, X-ray diffraction (XRD), and dynamic light scattering (DLS). The UD-AgNPs were evaluated against Gram-positive and Gram-negative bacteria, and their size, shape, and distribution were recorded. The average size of an NP was 19.401 nm. The zone of inhibition (ZOI) for 75 µL of UD-AgNPs against *Pseudomonas aeruginosa* (*P. aeruginosa*) was 21 ± 0.4 mm more than that of the control drug Ciprofloxacin (16 ± 10 mm). The minimum inhibitory concentration (MIC) was the lowest against *Escherichia coli* (*E. coli*) (36 ± 3 µg/mL) and *Staphylococcus* *epidermidis (S. epidermidis)* (38 ± 3 µg/mL). Moreover, the minimum bactericidal concentration (MBC) was the lowest against *E.* *coli* (75 ± 00 µg/mL) and *Enterococcus faecalis (E. faecalis* (83 ± 16 µg/mL). Thus, the UD-AgNPs synthesized using the ethyl acetate extract of UD can be used as a new antimicrobial drug.

## 1. Introduction

The benefits of medicinal plants have been intensively investigated for many centuries, as their natural components contain biologically active organic products, have few adverse effects, and are relatively low cost. It is estimated that plant products have provided the models for 50% of Western drugs [1]. *Urtica diocia* L. (UD), also known as stinging nettle, is an important medicinal plant that has been used since ancient times, and recent studies have reported that its leaves are a good food additive rich in medicinal value [2,3]. In addition, the crude extract of nettle plant leaves has been used to reduce inflammation [4] and maintain blood sugar levels in static conditions [5]. Multiple studies have also reported that the medicinal properties of UD include bleeding control and wound and burn healing. It also acts as a diuretic [6,7,8]. Moreover, crude UD extract has been reported as an antimicrobial agent against Gram-positive and Gram-negative microorganisms [9].

Antibiotic overdose as a result of an infectious disease treatment can lead to the emergence of multidrug-resistant (MDR) bacteria [10]. It has been documented that the Gram-positive bacterium *S. epidermidis* is MDR, which poses a healthcare management challenge [11]. This issue became a significant area of research into the formation of alternative neutral antibacterial agents with unique characteristics [12]. Advances in nanotechnology have allowed for the production of nanoparticles that show low toxicity against human cells and are reported to have promising bactericidal activity [13]. Phyto-nanotechnology, or plant-based nanomedicine, is used extensively in numerous fields in the form of nanoparticles (NPs) that enhance the activity of materials [14]. Recently, researchers have focused their attention on plant product-inspired metal NPs and their biomedical importance [15].

Among the different metal-based NPs, biosynthesized silver NPs (AgNPs) are well documented [16,17], biocompatible, and have few adverse effects [18]. Even a minimal amount of AgNPs has been shown to demonstrate antimicrobial [19], anticancer [20], antidiabetic [21], antiviral [22], and antioxidant properties [23]. Importantly, the synthesis of plant extract-based NPs is affordable, eco-friendly, and effective for formulating antimicrobial drugs [24]. The phytochemical screening of AgNPs containing Phoenix *dactylifera L*. leaf extract indicated polyphenols, flavonoids, and condensed tannins. These compounds play a vital role as a reducing and capping agent to inhibit particle growth and aggregation [25]. In addition, AgNPs from aqueous oregano plant leaf extract have been reported as non-infectious for sterile antimicrobial contact lenses [26].

The AgNPs synthesized with UD extract have been shown to protect the liver of cirrhotic rats [27]; similarly, AgNPs synthesized with *Urtica urens* are an effective nematicide against the root-knot nematode Meloidogyne [28]. A few studies have reported the antioxidant and antimicrobial effects of UD-AgNP aqueous extract [29,30] and methanol extract [31], indicating various effects such as the amount of AgNO_3_; however, reports on the size of the AgNPs and the concentration of UD-AgNPs in biological activities were absent. The current study aimed to conduct a green synthesis of AgNPs with ethyl acetate leaf extract of UD. The synthesized UD-AgNPs were characterized using UV–Vis, XRD, FTIR, SEM, TEM, EDX, and DLS analysis, and they were investigated for antimicrobial activity against Gram-positive and Gram-negative bacterial strains.

## 2. Materials and Methods

### 2.1. Collection and Preparation of Plant leaves

Fresh UD leaves, collected from the Riyadh region of Saudi Arabia, were rinsed with tap water and then with Milli Q water (ultrapure and deionized water purified by a Milli-Q water system, Merk, Darmstadt, Germany) to remove surface contaminants and other dust particles. The cleaned leaves were dried entirely at room temperature, after which 100 g of the dried leaves were milled into a fine powder [29].

### 2.2. UD Leaf Extraction

Ethyl acetate was used as the extraction solvent since it has a medium polarity with minimal cell toxicity. In brief, 10 mL of fine blended powder of UD leaves was poured into a Soxhlet extractor chamber. Then, 250 mL of ethyl acetate was placed in a round-bottomed flask and boiled at 77.1 °C [32]. This process was carried out in 24 h, and the ethyl acetate extracted compounds were retained.

### 2.3. Preparation of UD-AgNPs

Briefly, 1 mM of silver nitrate (AgNO_3_) dissolved in 250 mL of Milli Q water was added to 10 mL of ethyl acetate UD extract. The mixture was stirred continuously in a shaking incubator for 24 h and then monitored for interaction between the AgNO_3_ and the UD extract. The reaction (color changes) of the mixture started from 8 h onwards and turned completely dark brown within 24 h. The colored solution was placed in a rotary evaporator (Rotavapor R-215, Marshall Scientific, USA) at 45° C at 75 rpm to remove the solvent. The solution was centrifuged at 15,000 rpm for 15 min three times. The supernatant was discarded, and the NPs sedimented at the bottom of the centrifuge tube. The NP sediment tube was allowed to dry in an incubator at 40 °C for 2 h before collection [33,34]. The collected semisolid UD-AgNPs were kept at 4 °C for characterization and biological study [35].

### 2.4. Characterization

The absorption spectrum of the reaction mixture (AgNO_3_ + plant extract) after the stabilization of color was characterized by a UV–Vis spectrophotometer (LS 55; Perkin-Elmer, Rodgau, Germany). The size and shape of the UD-AgNPs were recorded using SEM (JEOL, Tokyo, Japan), TEM (JEOL), EDAX, XRD, and FTIR, according to the literature [14,15]. The size of the UD-AgNPs was determined by a Zetasizer DLS instrument (Malvern Instruments, Malvern, U.K).

### 2.5. Antimicrobial Evaluation

#### 2.5.1. Inhibition Study

The antibacterial activity of UD-AgNPs was evaluated against the following Gram-positive pathogens: *S. aureus* (ATCC^®^29213), *E. faecalis* (ATCC^®^29212), and *S. epidermidis* (ATCC^®^12228), as well as the following Gram-negative pathogens: *Klebsiella pneumoniae (K. pneumoniae)* (ATCC^®^700603*), P. aeruginosa* (ATCC^®^27853), and *E. coli* (ATCC^®^25922). In addition, their antimicrobial susceptibility was determined using Mueller–Hinton agar (MHA) plates [11,12]. Briefly, 19 g of MHA powder was mixed with 500 mL of distilled water and then sterilized in an autoclave at 121 °C for 15 min. Next, approximately 25 mL of the medium was poured into each plate, which had been inoculated with an adjusted bacterial concentration (10^8^ CFU/mL, 0.5 McFarland’s standard). Then, 40 mg of UD-AgNPs was dissolved in 20 mL of dimethyl sulfoxide (DMSO). Three different concentrations of UD-AgNPs in DMSO (25, 50, and 75 µL) were added to the plates. Ciprofloxacin (10 µL) was used as a positive control drug. The negative controls were DMSO, ethyl acetate UD extract, and AgNO_3_ (75 µL). The plates were incubated at 37 °C for 24 h. After incubation, the ZOI around the well, indicating the antimicrobial activity of the UD-AgNPs, was measured in mm [36].

#### 2.5.2. MIC

The MIC was determined by a resazurin-based assay using UD-AgNP concentrations ranging from 15 to 50 µg/mL with an adjusted bacterial concentration (10^8^ CFU/mL, 0.5 McFarland’s standard). Three 96-well plates were used for the MIC evaluation. In the first plate, the first six columns were filled with 190 µL of Mueller–Hinton Broth (MHB) and 10 µL of each type of pathogen as a positive control. In comparison, the other six columns of the first plate and the second plate were treated with serial dilutions of UD-AgNPs, with concentrations ranging from 15 to 50 µg/mL (15, 20, 25, 30, 35, 40, 45, and 50 µg/mL) and 10 µL of each type of pathogen. In the third plate, three columns were filled with 150 µL of MHB and used as a negative control. After incubation for 24 h, 15 µL of 0.02% resazurin solution (5 mg in 25 mL of distilled water) was added to each well of the microtiter plates, and the plates were further incubated at 37 °C for 4 h. Color changes were recorded, and the MIC values were determined. The MIC was considered the lowest concentration of the UD-AgNPs that inhibited bacterial growth [37].

#### 2.5.3. MBC

The MBC was determined according to the protocol of the Clinical and Laboratory Standards Institute, and MHA plates were used for the MBC experiments. Briefly, 10 µL of pathogen was inoculated onto each plate, and then different amounts of UD-AgNPs (25, 50, 75, 100, 150, 200, 250, and 500 µL) of a mixture of 40 mg of UD-AgNPs dissolved in 20 mL of DMSO were applied to the agar plates. The plates were incubated at 37 °C for 24 h. The plates started with no visible bacterial growth in specific concentrations considered as MBC. The MBC value of the UD-AgNPs for each tested microorganism was determined [38].

### 2.6. Statistical Analysis

The experimental data were analyzed using one-way ANOVA. The data were represented as the mean ± standard deviation (SD). The experiments were performed with a level of significance of *p* ≤ 0.05.

## 3. Results

### 3.1. Characterizations

#### 3.1.1. Visual Observation

**Figure 1:**
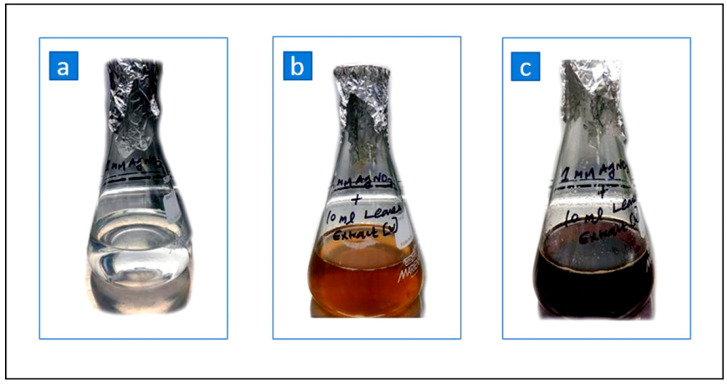
Ethyl acetate extract of UD leaves mediated the preparation of AgNPs. (**a**) Silver nitrate solution; (**b**) mixture of plant extract and silver nitrate; (**c**) formation of AgNPs.

The combination of the ethyl acetate extract of UD leaves and the AgNO_3_ solution produced UD-AgNPs after 8 h. Initially, the AgNO_3_ solution was colorless, and the mixture of AgNO_3_ and the plant extract was yellow, as shown in Figure 1a,b. After stirring for 8 h, the mixture of the ethyl acetate extract of UD leaves and AgNO_3_ solution turned dark brown, indicating the formation of UD-AgNPs (Figure 1c). The change in color was due to the interaction between the UD leaf extract and the formation of capping and reducing agents (Ag^+^ to Ag).

#### 3.1.2. UV–vis Spectral Study

**Figure 2:**
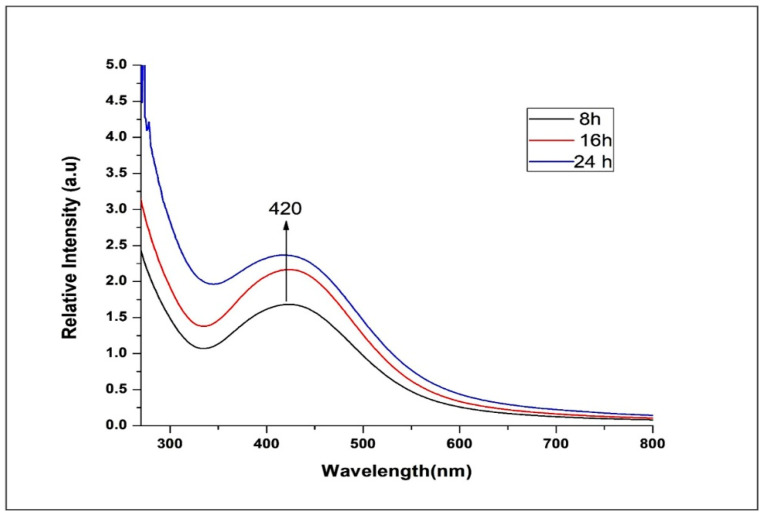
UV–vis spectrum of the UD-AgNPs recorded at different time points.

The UV–vis spectral images at different time points (8, 16, and 24 h) showed the progression of NP formation. An absorbance peak was noted at 420 nm (Figure 2). Surface plasmon resonance experiments indicated that the solution of UD-AgNPs was stable for 24 h.

#### 3.1.3. FTIR Spectroscopy

**Figure 3:**
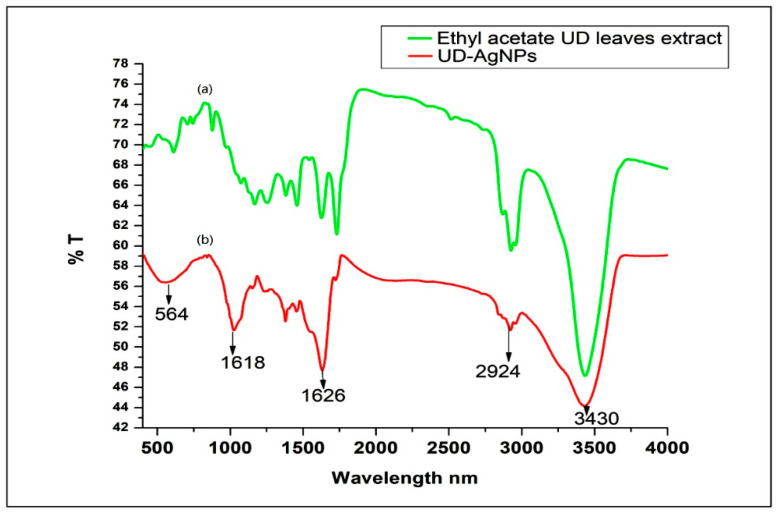
FTIR spectrum. (**a**) Ethyl acetate UD leaves extract; (**b**) UD-AgNPs with different functional groups.

The FTIR spectrum indicated the ethyl acetate leaf extract alone (Figure 3a) and the synthesized UD-AgNPs (Figure 3b). The FTIR absorption spectra of acetate leaf extract alone peaked at 3433, 2925, 1730, 1624, 1166, and 611 cm^−1^. In contrast, the synthesized UD-AgNPs absorption peaks were shifted slightly at 3430, 2924, 1626, 1018, and 564 cm^−1^. The strong absorption peaks at 3430 cm^−1^ corresponded to the N–H stretch (primary amine). In addition, a long, narrow band was seen at 2924 cm^−1^, indicating the presence of C–H stretching (alkane). Strong bands were also noted at 1626 cm^−1^, representing C=C stretching (alkene). Moreover, bands were formed at 1018 cm^−1^, corresponding to C–N stretching (amines). Another peak at 564 cm^−1^ belonged to the C–I or C–Br group of the compounds. The similar spectral features for acetate leaf extract alone and the synthesizeVirulence determinants of uropathogenic Escherichia coli in fecal strains from intestinal infections and healthy individuals. UD-AgNPs may have indicated the presence of the identified phytochemicals in the synthesized UD-AgNPs.

#### 3.1.4. XRD Pattern

**Figure 4:**
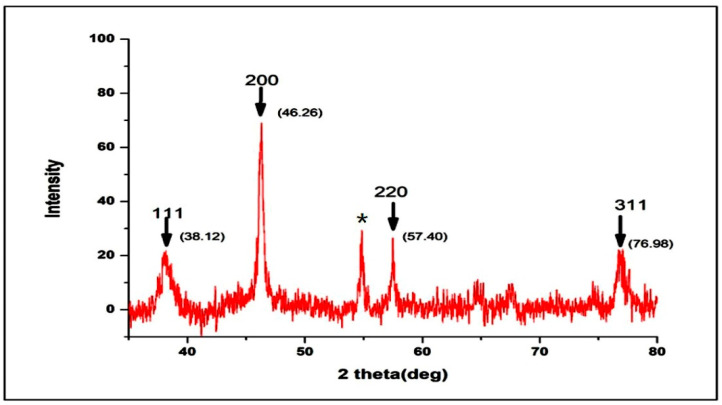
XRD pattern of the UD-AgNPs with peak values.

The UD-AgNPs were studied using XRD, and the results are shown in Figure 4. The XRD pattern exhibited peaks at the two theta values of 38.12, 46.26, 57.40, and 76.98°, corresponding to the *hkl* plane Bragg reflections 111, 200, 220, and 311, respectively. The XRD spectrum confirmed that the synthesized UD-AgNPs had a face-centered cubic structure. The unassigned peak (*), observed at 55°, could have been caused by bioorganic compounds in the synthesized NPs.

#### 3.1.5. DLS Analysis

**Figure 5:**
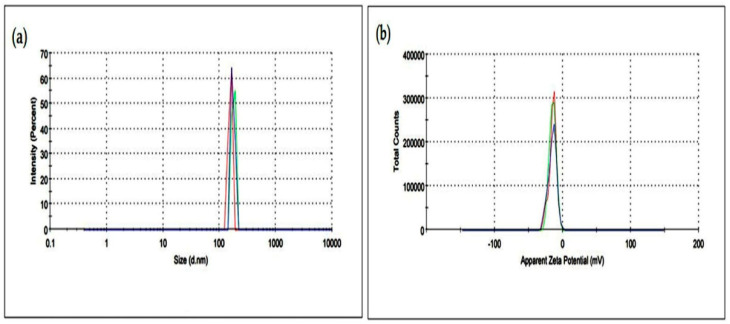
DLS Zetasizer analysis (**a**) Size distribution of the synthesized UD-AgNPs (**b**) Zeta potential of the synthesized UD-AgNPs.

The DLS Zetasizer was used to determine the size distribution and the surface intensity of the synthesized UD-AgNPs (Figure 5a–b). The size distribution was measured based on the intensity fluctuation yields of the rate of Brownian motion (Figure 5a). The DLS result exhibited the average peak intensity values in the hydrodynamic diameter distribution with relatively homogenous distribution in the polydispersity index (PDI: 0.47 ± 0.31).

The zeta potential result indicated that the distribution of NPs was equal, without any aggregation of UD-AgNPs (Figure 5b). The negative value (−15.5 ± 0.6 mV) of the zeta potential confirmed the size distribution and low aggregation of the synthesized UD-AgNPs, which was important for understanding their stability.

#### 3.1.6. EDAX Analysis

**Figure 6:**
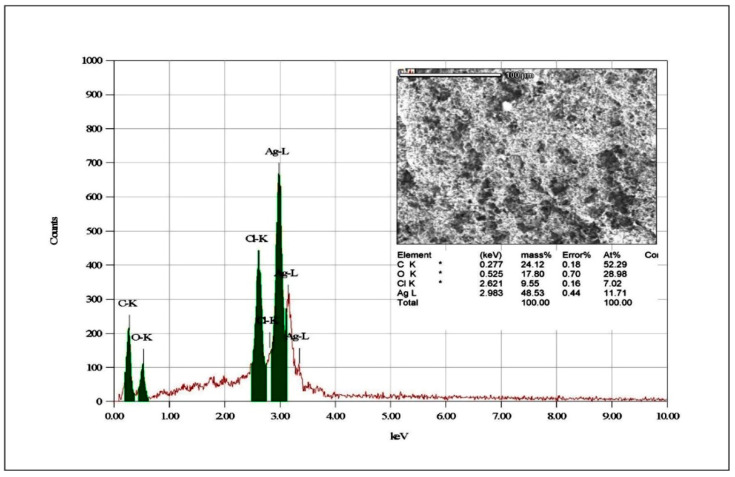
EADX spectrum observed elemental distribution Ag–L, C–K, O–K and Cl–K from synthesized UD-AgNPs.

As depicted in Figure 6, the EDAX for the elemental distribution in the synthesized UD-AgNPs shows that the elemental composition of Ag–L (48.53% mass) was higher than that of the other components: C–K (24.12% mass), O–K (17.80% mass), and Cl–K (9.55% mass). These results demonstrated that the AgNPs synthesized with UD-based NPs were pure.

#### 3.1.7. SEM and TEM Images

**Figure 7:**
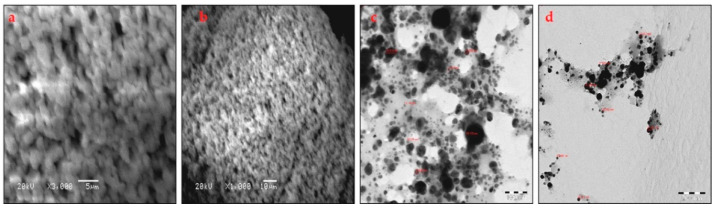
(**a**–**d**) SEM with different magnifications (**a**) 5 µm and (**b**) 10 µm; TEM images with different magnifications (**c**) 100 nm and (**d**) 200 nm exhibiting the formation of UD-AgNPs.

The SEM images of the UD-AgNPs shown in Figure 7a,b reveal that the UD-AgNPs were spherical and did not aggregate. The images confirmed the formation of AgNPs. The TEM images of the UD-AgNPs depicted in Figure 7c,d showed that the UD-AgNPs had a spherical shape ranging from 8.2 to 56.1 nm, with an average size of 19.4 nm.

### 3.2. Antimicrobial Susceptibility

**Figure 8:**
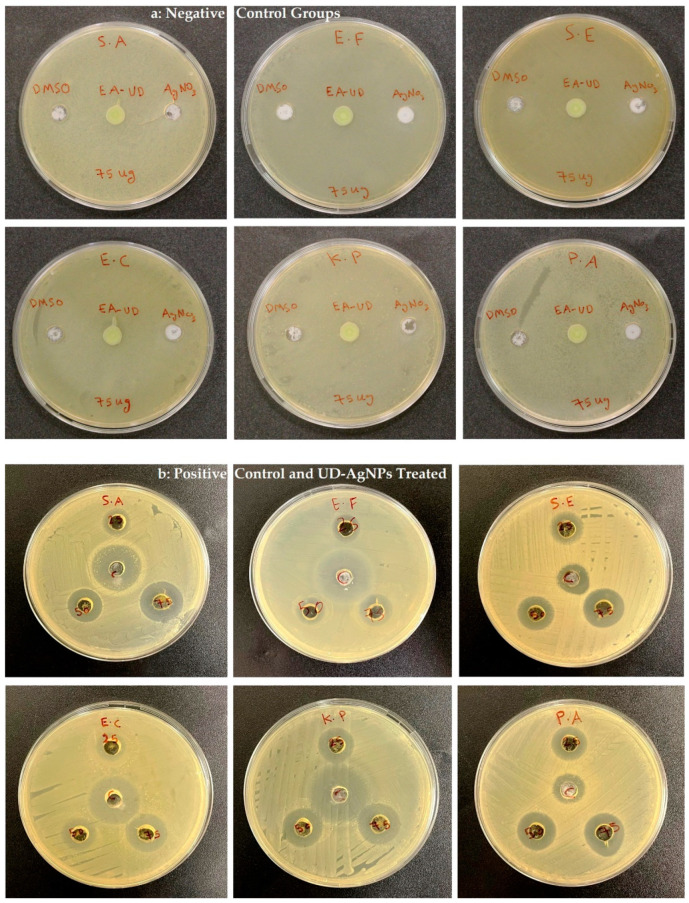
ZOI (**a**) control groups (DMSO, UD extract, and AgNO_3_) and (**b**) synthesized UD-AgNPs against *S. aureus*, *E. faecalis*, *S. epidermidis*, *E. coli*, *K. pneumoniae*, and *P. aeruginosa*.

The antimicrobial susceptibility of the UD-AgNPs against Gram-positive and Gram-negative bacteria was tested at 25, 50, and 75 µL. Ciprofloxacin (10 µL) was used as a positive control, and the negative control test was performed using DMSO, ethyl acetate UD leaf extract, and AgNO_3_ (75 µL). No record was found related to ZOI in the tested microorganisms (Figure 8a). The ZOI results of Gram-positive bacteria *S. aureus*, *E. faecalis*, and *S. epidermidis*, as well as those of the Gram-negative bacteria *E. coli*, *K. pneumoniae*, and *P. aeruginosa* are given in Figure 8b. The ZOI values are shown in Table 1. Compared to the other Gram-positive bacteria, the maximum ZOI was recorded for *S. epidermidis* (19 ± 1 mm). Among the Gram-negative bacteria, *P. aeruginosa* exhibited the highest ZOI value (21.6 ± 0.4 mm) (Table 1).

#### 3.2.1. MIC Analysis

**Figure 9:**
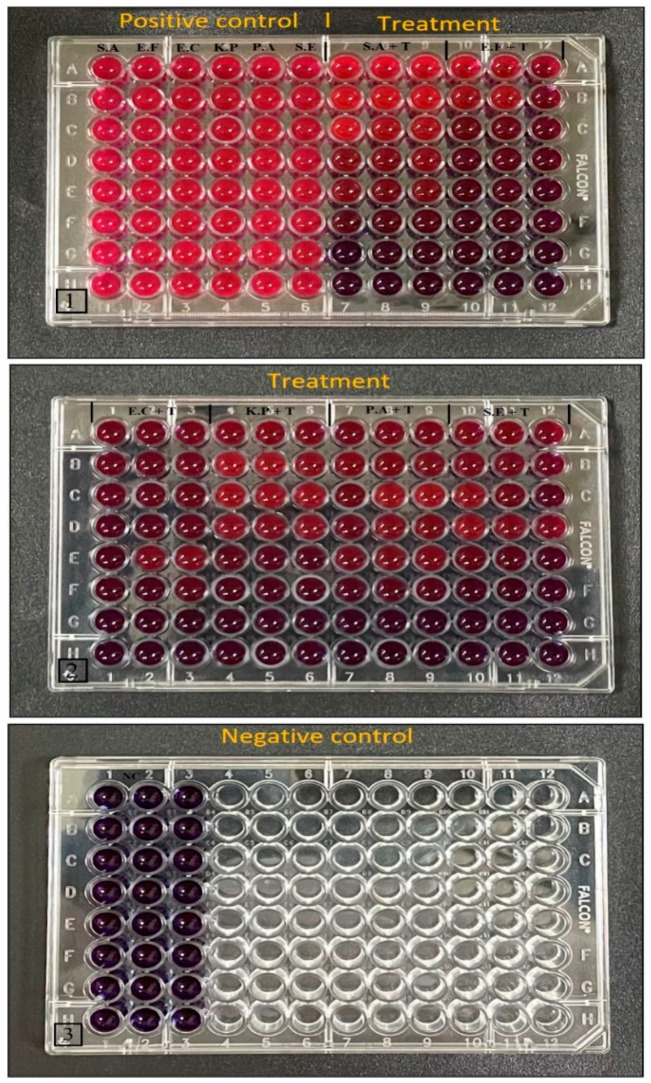
Resazurin Plate assay showing the MIC values of the UD-AgNPs against *S. aureus*, *E. faecalis*, *S. epidermidis*, *E. coli*, *K. pneumoniae*, and *P. aeruginosa*.

The MIC values of the UD-AgNPs against the Gram-positive bacteria *S. aureus*, *E. faecalis*, and *S. epidermidis,* as well as the Gram-negative bacteria *E. coli*, *K. pneumoniae*, and *P. aeruginosa,* were determined by assaying various concentrations of the UD-AgNPs (15, 20, 25, 30, 35, 40, 45, and 50 µg/mL). Three 96-well plates were used to assay the six microorganisms with the positive control (10 µL of each type of pathogen) and treatment (UD-AgNPs) incubated for 24 h at 37 °C. Resazurin dye (negative control) was used as a precursor to discriminate the actual MIC value of each tested organism. In this assay, the MIC values for each organism were based on color changes (Figure 9). The MIC values of the synthesized UD-AgNPs against the pathogens tested are shown in Table 2

#### 3.2.2. MBC Analysis

The MBC values for both Gram-positive bacteria and Gram-negative bacteria are shown in Table 2. The results indicated that the UD-AgNPs indeed inhibited the growth of the tested pathogens.

## 4. Discussion

The current research focused on synthesizing AgNPs by using the medicinal plant UD, the leaf extract of which acted as a capping and reducing agent (AgNO_3_ into AgNPs) and presented numerous phytochemical compounds [25,26]. The formation of AgNPs was confirmed by changes in the color of the mixture [39] and the UV–vis spectral peak at 420–460 nm [40].

The FTIR spectrum showed strong absorption peaks corresponding to N–H and C–N; these amino groups played a role in the conversion process of Ag^+^ to Ag. The presence of C=C and C–H bonds revealed that the alkenyl groups were involved in the strengthening of AgNPs. The alkenyl group strengthened the AgNPs as a capping agent, which could selectively bind to different types of small plane surfaces to change their specific surface-free energies. The capping agents were involved in numerous important functions, such as preventing the agglomeration of nanoparticles and reducing toxicity. Moreover, alkenyl group molecules enhanced AgNPs in bacterial activities [41,42]. Another peak at 564 cm^−1^ indicated the presence of a C–Br group, the secondary structure of proteins that interacted with the NPs [29,43]. The reported wave numbers indicating signal stretching and vibrational bending of the peaks may have been derived from phytoconstituents such as flavonoids, terpenoids, alkaloids, and soluble proteins present in plant extracts. These compounds may have been responsible for chelating and capping in the bioreduction process [44,45]. The peaks indexed to the 111, 200, and 220 planes corresponded to the cubic structure of silver, and the Debye–Scherrer equation confirmed the small and crystalline NPs [46]. Because the synthesized NPs were small, their diffusion was very fast, as reported previously [47]. According to the DLS (PDI: 0.47 ± 031) and ZP results (−15.5 ± 0.6 mV), the size and surface charge of the NPs confirmed their therapeutic potential. These two traits influenced their kinetics and interaction with cellular and biological membranes, resulting in their efficacy [45].

Synthesized UD-AgNPs showed Ag–L (48.53% mass) was more abundant than other components such as C–K, O–K, and Cl–K by EDAX, as previously reported [48]. The presence of a trace amount of oxygen (17.80% mass) in the UD-AgNPs confirmed the roles played by the UD phytochemicals in the reduction in metal ions, capping agents, and NP stability. Based on these characterizations, it was clear that the synthesized UD-AgNPs were small, spherical, and not agglomerated. Size-related properties influenced the NP mode of action [49,50,51].

In the present study, the antimicrobial potency of the synthesized UD-AgNPs was studied against both Gram-positive and Gram-negative pathogens. Of the Gram-positive bacteria tested, *S. epidermidis* showed the greatest ZOI, indicating its high susceptibility to the AgNPs. The earlier study showed that the ZOI was greater for *S. epidermidis,* which was more sensitive to AgNPs [52]. In the case of the other pathogens (*S. aureus* and *E. faecalis*), they had a decreased uptake of UD-AgNPs, increased efflux pump activity, and increased drug-degrading enzymatic activity, which may be the reasons for their lower susceptibility to the UD-AgNPs.

Among the three Gram-negative pathogens tested, *P. aeruginosa* exhibited the largest ZOI, followed by *E. coli* and *K. pneumoniae*, indicating sensitivity to the UD-AgNPs. The internalization of the AgNPs in *P. aeruginosa* was greater than that of the other pathogens. Previous researchers have reported that *P. aeruginosa* is highly drug-resistant, which limits the use of many antibiotics. However, it is very interesting to note that this pathogen is highly sensitive to UD-AgNPs, as reported for a few other biosynthesized AgNPs [53,54]. The Gram-negative bacteria *E. coli* and *K. pneumoniae* have been found to be resistant to many drugs, but they were sensitive to the synthesized AgNPs. It has been reported that AgNPs induce a triclosan-like bactericidal mechanism to inhibit type II fatty-acid biosynthesis and generates oxidative stress in *K. pneumoniae*, which has shown antibacterial activity in multidrug-resistant *K. pneumoniae* [37]. Like *K. pneumoniae* with extended-spectrum beta-lactamase, *E. coli* is very sensitive to NPs, and the NPs inhibit the bacterium by making the cell shrink and impairing its membrane integrity [55]. In the present study, sensitivity studies showed that the Gram-negative bacteria were more susceptible to the action of AgNPs. It has been reported that the difference in cell wall components facilitates the entry of released ions from AgNPs more into the cells of Gram-negative than Gram-positive bacteria [56].

The MIC and MBC values reflected the susceptibility of the microbes to UD-AgNPs. The high MIC values against *E*. *coli* and *S. epidermidis* and MBC against *E.* *coli* and *E. faecalis* were due to the action of lipopolysaccharides against the trapped AgNPs, as reported previously [57]. This finding reflected the strong toxicity of UD-AgNPs against the microorganism [58]. Therefore, AgNPs can be used as nanobactericides and drug carriers, as reported [57]. Hence, the outcome of this study confirmed that the ethyl acetate-mediated synthesized UD-AgNPs acted as nanobactericides, meaning that they can penetrate bacterial envelopes to damage the cell membrane structure causing oxidative stress [59,60,61].

## 5. Conclusions

UD is a medicinal plant, and its leaves possess numerous medicinal properties, including controlling cardiovascular disorders and glucose homeostasis. Using the ethyl acetate extracts of UD leaves, AgNPs were synthesized in this study. The characterization of the synthesized NPs showed that the particles were spherical and small, and since small NPs have favorable therapeutic actions, the currently synthesized NPs have promising prospects for development as drugs. This characterization study further confirmed the presence of bioactive compounds in the leaves and that they became incorporated into the AgNPs. Such morphometric changes and functional adaptations promote the attachment of the AgNPs to the cell wall of the pathogen, which then internalizes the NPs, which in turn alter the internal environment of the bacterial cell and induce oxidative stress, causing changes in the DNA and the cellular enzymes that lead to bacterial cell death or functional inhibition. The ZOI, MIC, and MBC results showed that the synthesized NPs had bactericidal action against both Gram-positive and Gram-negative bacteria. Thus, future research should focus on the activity of the newly synthesized UD-AgNPs towards multidrug resistance and extended-spectrum beta-lactamase-containing and methicillin-resistant bacteria.

## Figures and Tables

**Table 1:** The zone of inhibition observed for different bacterial isolates and different amounts of UD-AgNPs.

Zone of Inhibition (mm)				
Bacteria	25 uL (Mean ± SD)	50 uL (Mean ± SD)	75 uL (Mean± SD)	Control
*S. aureus*	13.3 ± 0.7	16.6 ± 0.4	21.3 ± 0.7	24 ± 1
*E. faecalis*	12.6 ± 0.4	14 ± 1	18.6 ± 0.4	27 ± 1
*S.* *epidermidis*	14.3 ± 0.7	17.6 ± 0.6	19 ± 1	15.6 ± 0.6
*K. pneumoniae*	16.3 ± 0.7	17.3 ± 0.7	19.6 ± 0.6	26 ± 1
*P. aeruginosa*	16 ± 1	18.6 ± 0.4	21.6 ± 0.4	16 ± 1
*E. coli*	13.6 ± 0.6	17 ± 1	19.6 ± 0.6	24.3 ± 0.7

**Table 2:** Minimum inhibitory concentrations (MICs) and minimum bactericidal concentration (MBC) of UD-AgNPs against Gram-positive and Gram-negative bacteria.

Bacteria	MIC (µg/mL) (Mean ± SD)	MBC (µg/mL) (Mean ± SD)
*S. aureus*	40 ± 0	92 ± 17
*S. epidermidis*	38 ± 3	233 ± 33
*E. faecalis*	42 ± 8	83 ± 17
*K. pneumoniae*	40 ± 0	333 ± 33
*P. aeruginosa*	40 ± 0	133.± 33
*E. coli*	37± 3	75 ± 00

## Data Availability

The data presented in this study are available on request from the corresponding author.
